# Carbon storage in mountain cloud forest communities, Jalpan de Serra, Querétaro, México

**DOI:** 10.1186/s13021-025-00324-1

**Published:** 2025-10-10

**Authors:** Fuentes-Romero Elizabeth, García Calderón Norma Eugenia, Sedov Sergey, López-Binnqüist Citlalli, Noé Velázquez-Rosas

**Affiliations:** 1 Unidad Multidisciplinaria de Docencia e Investigación, Facultad de Ciencias, Universidad Nacional Autónoma de México, Juriquilla Querétaro, México; 2 Centro de Investigaciones Tropicales, Universidad Veracruzana, Xalapa Veracruz, México; 3 Instituto de Geología, Universidad Nacional Autónoma de México, Ciudad de Mexico, México

**Keywords:** Carbon stocks, Above-ground biomass, Organic horizon, Degraded forests

## Abstract

**Background:**

Mountain cloud forests (MCF) are vulnerable ecosystems that harbor considerable biodiversity and are essential carbon regulators. However, information is scarce on the carbon storage potential and its patterns of variability across the conservation gradient in these forests. This study determined the carbon storage potential, the contribution of different pools, and their relationship with the degree of forest and soil conservation.

**Results:**

The organic carbon storage of the communities ranged from 145.9 to 279 Mg C ha^−1^. Soil was the primary pool (68.08–198.1 Mg C ha^−1^), followed by above-ground biomass (42.87 – 116.74 Mg C ha^−1^), while the contribution of litter and roots was less. The contribution of above-ground biomass to the carbon stock was low due to the level of timber and fuelwood extraction present in these communities. The high carbon storage potential of the soil pool is determined by the presence of the O horizon, with a thickness of 8–10 cm, forming mull-type humus and a deep organo-mineral surface horizon with a high carbon content > 10 g kg^−1^, and with varying degrees of humification. The formation of clay-humus complexes maintains carbon stabilization and the formation of deep surface horizons (between 20 and 38 cm deep).

**Conclusion:**

The results show that the carbon sequestration potential of the MCF is found in the soil associated with the organic horizons that develop at the surface and the presence of deep A horizons with high carbon content. The conservation of these layers, despite forest management, reflected in the aerial biomass, demonstrates the resilience of the soil due to carbon stabilization, attributed to the composition of resistant organic compounds and the formation of clay-humus complexes, which reduce the impact of degradation from erosion. This indicates that the conditions of the MCF still sustain the ecological and biogeochemical processes that support carbon sequestration and are regulated by the conservation policies of the Sierra Gorda Biosphere Reserve, Querétaro, Mexico.

## Background

At a global level, MCFs are important organic carbon (OC) sinks, and the sources and processes that regulate their dynamics have a high potential for forest carbon sequestration [1]. Carbon dynamics in these ecosystems depend on the mechanisms of capture, emission, and stabilization in the different pools [2], with mean residence time (MRT) depending on environmental conditions [3]. The C in above-ground biomass, root biomass, litter, necromass, and soil depends on interactions between pools and environmental factors; in particular, temperature and humidity [4–6]. C in litter, plant biomass, and foliage are considered to have a short MRT (weeks/months), whereas MRT of the structural part of litter can vary from 1 to 3 years and wood biomass and structural molecules of passive intermediate organic matter have an MRT of > 10^3^ years [7].

Primary neotropical forests store between 141 and 571 Mg ha^−1^, while temperate forests store about 179 ± 6 Pg C of global carbon [8]. Carbon storage in plant communities is strongly related to the degree of degradation and succession, as well as to changes in rainfall, cloud, and fog patterns due to climate change [9, 10]. The changes in OC sink dynamics are associated with disturbance and fragmentation regimes in ecosystems due to vegetation harvesting, fires, and land use change, among others [11, 12]. This leads to a shift in species composition [13, 14] and the structure and assembly of functional groups, affecting tree density and basal area in the early stages of the disturbance [10, 15]. Soil organic carbon (SOC) sinks are related to the accumulation, quality, and diversity of residues, as well as to the population of decomposer organisms that decrease the thickness of the organic horizon [15, 16]. Alterations in the decomposer community affect the carbon storage potential, as well as CO_2_ production and emission, which is reflected in the decomposition of organic residues in litter and nitrogen fixation [10, 17]. Ecosystem degradation has caused the reduction of OC sinks by about 15–52 Mg ha^−1^ due to land use change and forest fires [18, 19]. Despite this, secondary and managed forests have recorded stocks of up to 2.2 Pg SOC, sequestered in the O and surface soil horizons [20, 21].

Forest soil has played an essential role as a carbon pool within the ecosystem, as they have a high global sequestration potential of approximately 383 Pg C, which represents 44% of the total carbon stocks in terrestrial ecosystems [11, 20]. SOC stabilization is influenced by the flux of CO_2_ from the soil to the atmosphere and with the rate of decomposition of organic matter [11, 12], which depends on moisture and temperature regulating heterotrophic respiration [6, 21]. In MCFs, CO_2_ emission decreases due to increased soil moisture and decreased oxygen in the soil [22]. In addition, there is a stabilization of SOC sinks with the solid phase of the soil due to the formation of complex mineral bodies [23–25].

MCFs are considered a priority for conservation because of the environmental services they provide and the biodiversity they protect [9, 26–28]. However, these forests are at risk worldwide due to their limited distribution on a global scale, the effects of climate change, and anthropogenic pressure, particularly impacts related to agricultural and livestock management [26, 27]. In Mexico, MCFs represent less than 0.5% of the total country area and are considered a highly diverse vegetation type because they host 10% of the nation’s plant richness and 30% of endemic species [29, 30]. One of their relevant functions is their high capacity to fix CO_2_, determined by their structure and the environmental conditions in which they develop [22, 24, 31]. Diverse studies on MCF demonstrate their importance for C storage; on average, they can reach 384.16 Mg C ha^−1^ [32], while temperate coniferous forests can store from 67 to 177 Mg C ha^−1^ [4, 33, 34]. The OC pools in MCF are relevant considering that above-ground biomass reaches up to 56.7 Mg C ha^−1^; whereas the soil has a larger storage potential between 158.2 and 222.5 Mg C ha^−1^, while in the O horizon 2.2–3.1 Mg C ha^−1^ have been recorded [32].

OC storage in MCF has been related to the increase in net primary productivity, associated with the ratio of the increase in biomass production to the increase in net primary productivity [35]. In particular, the high OC storage potential of these forests is due to the trees, the main structural elements of the community [23, 24], the nutrient composition of the leaves [36], and the nutrient balance between soil and plants, which leads to changes in the biogeochemical cycles associated with nutrient use efficiency, and which substantially increases competition between species, especially for nitrogen [37]. The storage and stabilization of C in the soil are related to the MRT of the pools, associated with the low decomposition rate of soil organic matter, the complexity of organic molecules, and the thickness of the forest organic horizon [24]. This affects the decomposition of MOS, as well as the production and emission of CO [22], due to low dissolved oxygen levels, soil moisture saturation, and high rainfall. These factors influence the community of decomposer microorganisms, leading to a reduction in mass loss in the organic layers by up to 70% [38–40].

The quantity and quality of organic residues influence carbon accumulation in the soil. The presence of compounds such as lignin, nitrogen levels, the C:N ratio, and non-polar compounds acts as chemical controllers of decomposition. These components confer primary recalcitrance due to the complex mixtures in the soil humus. The accumulation of microbial products, coal, and the formation of humic polymers contribute to secondary recalcitrance [38–40]. SOC storage and stabilization are also associated with clay-humus complexes and organic compounds with different degrees of evolution [23, 25]. Polymerization to high aromaticity compounds promotes dominance of humic substances [32]. The accumulation of these compounds at depth has been associated with translocation at sites with high precipitation and soil washout rates [25]. SOC stabilization is also related to sequestration in stable macro- and mesoaggregates, as well as a significant proportion of particulate soil organic matter (SOM), especially in surface soil horizons [41].

The tropical forest is recognized for its ability to capture carbon due to its high diversity and productivity. It retains 40% of terrestrial carbon and sequesters between 2 and 5 ton ha^−1^ year of CO_2_. Therefore, these ecosystems are vital for carbon sequestration in vegetation and soil, allowing for long-term regulation [42]. The carbon stored in tropical forests can be lost due to human activities, thereby risking their ability to regulate and sequester carbon [43]. The deforestation rate of tropical forests, including MCFs, globally ranges from −1.1% to −13% per year, due to the expansion of agricultural frontiers, wildfires, logging, and conversion to pasture [43–45]. The extraction of wood for fuel affects plant communities [46], leading to changes in their structure and a decline in community composition. Human activity impacts the carbon sequestration potential of forests.

The MCFs have significant ecological importance, as they protect biodiversity and contain a considerable range of specialized, endemic plants and animals adapted to the microclimatic conditions of the ecosystem [47]. Additionally, its importance lies in regulating the water captured from clouds and fog that maintains the hydrological cycle in rivers. Its role in carbon capture is crucial due to the global contribution of plant biomass (271–254 ton ha^−1^), mainly because of the presence of specific species that maintain carbon sequestration, despite slight variations in storage related to altitude and relief conditions [42, 48]. Likewise, the contribution of detritus that accumulates on the soil surface and the high potential of the soil to store carbon, due to the accumulation and stabilization of organic matter under conditions of high humidity and low oxygen [22, 25, 32, 49, 50], supports the idea that the importance of the MCF in carbon sequestration is much higher than previously assumed, making it crucial to conserve them against climate change and degradation from management practices. The Carbon pool depends on biophysical and geochemical processes and management practices that determine C sequestration at the landscape level. Therefore, their assessment and spatial monitoring of carbon dynamics is essential to determine the potential and change in the terrestrial carbon pool, which can ensure greenhouse gas mitigation processes through carbon sequestration [3, 6]. Considering the importance of MCFs in the carbon cycle and the level of degradation they are currently facing [26, 27], it is a priority to understand the carbon dynamics of these forests. This ecological and edaphic study aims to determine the contribution of different carbon pools within the ecosystem due to the importance of the tropical montane cloud forest (MCF) in carbon sequestration by the tree layer and soil [32, 48–50]. Specifically, we determined (i) the composition, structure, and carbon storage of the above-ground biomass; (ii) the contribution of the carbon pool by the soil organic layers, roots, and the carbon stored in the soil down to its maximum depth of development; and (iii) to discuss the importance of these reservoirs in relation to the overall carbon storage potential of MCF and their relation to the degree of conservation.

## Methods

### Site description

The study area was located in the micro-basin Valle Verde-Ojo de Agua de San Francisco, Jalpan de Serra, Querétaro, between 21°34′32.90"N and 99°12′44.36"W; 21°34′33.95" N and 99° 9′39.07"W. This micro-basin is part of the Sierra Madre Oriental, subprovince of Carso Huasteco. The landscape is tectonic-structural with mountains and hillsides with complex and steep slopes, as well as intermontane depressions. In addition, there is karst landscape with the formation of sinkholes, chasms, caverns, and poljes [51]. The soil is formed from calcareous material from the El Soyatal and El Doctor formations associated with granodioritic intrusive igneous materials from the Lower Cretaceous (114–95 Ma) [52]. In addition, there is soil formed from the calcareous-sandstone association and shales of the Mexcala Formation, from the Middle and Upper Cretaceous (95–65 Ma) [52, 53].

The climate of the region is temperate sub-humid with summer rainfall Cb(w2)(i´gw”), according to the Köppen classification modified by García [54]. Average annual rainfall is between 1000 and 1200 mm, with the highest rainfall intensity > 250 mm day^−1^. The average annual temperature is ~ 18 °C. The edaphodiversity is formed by Distric Litic Leptosols associated with Haplic Luvisols (Leptic) and with the sequence Albic Fragic Luvisols (Calcaric, Cutanic) and Umbric Alisols (Cutanic, Humic) [25].

The vegetation of the region is transitional between temperate pine, oak, and mountain cloud forests in an altitudinal gradient of 800–1400 m [55, 56]. The MCF has an area of approximately 54 km.^2^ and represents about 0.5% of the state’s territory. It is distributed in Pinal de Amoles, Jalpan de Serra, Landa de Matamoros and San Joaquín, Querétaro. The state of succession of the plant communities is heterogeneous, with the areas of greatest conservation located in Landa de Matamoros in a gradient between 1000 and 1400 m, where the community reaches its maximum level of structure and diversity with three levels of tree strata over 30 m in height [57]. The dominant tree species are *Quercus affinis, Q. germana, Q. microphylla, Q. polymorpha, Q. sartorii, Q. xalapensis, Robinsonella discolor y Ulmus mexicana, Aphananthe monoica, Cinnamomum effusum, Inga huastecana, Photinia mexicana, Reevesia clarkii, Sapindus saponaria, Senna racemosa* and *Wimmeria concolor* [56, 57].

The zone is managed for conservation and sustainable use, as it is part of the buffer zone of the Sierra Gorda Biosphere Reserve, Querétaro (RBSGQ)). In addition, subzones have been established for agroforestry, livestock, and firewood extraction, as well as cattle exclusion zones to maintain the ecological integrity of the communities [58].

### Forest sites

**Fig. 1:**
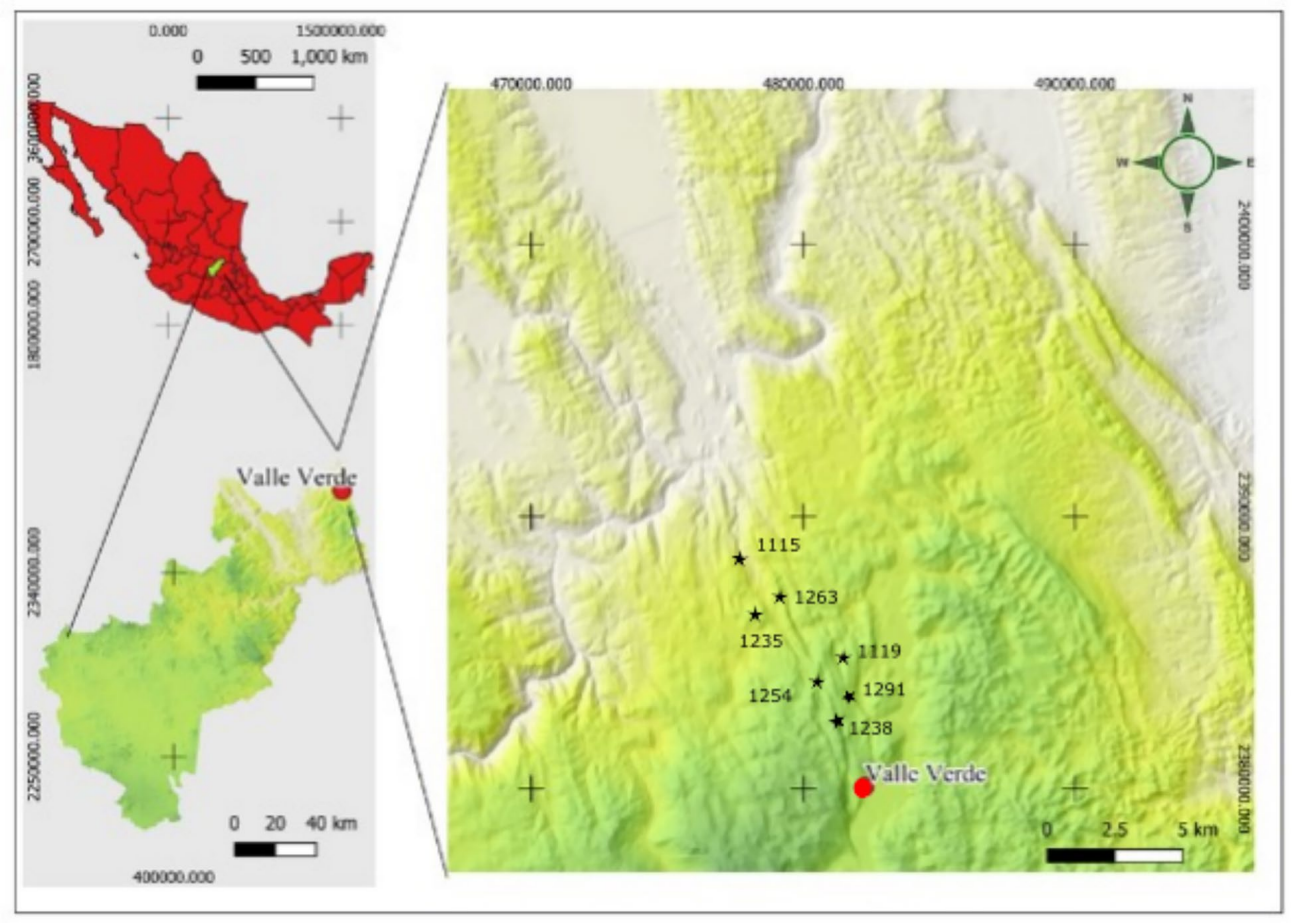
Location of cloud forest communities in the micro-basin of Valle Verde—Ojo de Agua de San Francisco, Jalpan de Serra, Querétaro

Seven plant communities with different conservation status were selected within the MCF. The sites were established within a sequence of medium and high hillsides, on steep slopes with a northwest-northeast orientation at an altitude between 1115 and 1291 m asl (Fig. 1).

### Survey of tree composition, structure, biomass, and carbon stock

In each of the sites, the tree component was sampled in 10 plots of 10 × 10 m (0.1 ha). Each of the plots were 20 m apart from each other and were located in three positions on the slope, three plots in the upper part, four in the middle position, and three in the lower part. In each sampling unit, the diameter at breast height (DBH, 1.3 m from the ground) was measured for trees with a DBH > 2.5 cm. In addition, botanical samples of each morphospecies identified were collected and herborized to determine their taxonomic identity. Stem density, basal area, and Shannon’s diversity index were calculated for each community [59, 60]. The estimation of above-ground biomass, in each of the species, was carried out by a non-destructive method, using allometric formula for the tree biomass developed by Acosta-Mireles et al. [61]:

$\text{ln}(Y)=-2.194 + 2.364\text{ ln }(\text{X})$, where Y = above-ground biomass and X = DBH.

For this calculation, all individuals with a DBH > 2.5 cm were considered. The biomass of each site corresponded to the sum of the biomass of all species.

In order, to determine the influence of plant size on aboveground biomass, at each site all stems were classified into four diametric categories (2.5–10.0, 10.1–30; 30.1–60.0 and > 60.1 cm). Subsequently, the estimated biomass for each stem in each diameter category was summed.

To calculate the aboveground biomass carbon stock at each site, the biomass was transformed to stores of C applying the conversion factor of 0.5 [62]. The total root biomass (fine, medium, and coarse) was calculated using root biomass to aerial biomass ratio of 19% [32]. Subsequently, it was transformed with the conversion factor of 0.5 [63] to obtain the root carbon stock.

### Determination of the carbon stock in the organic horizon and soil

In each plant community, a systematic design was established using a geo-referenced point clock method [64], within a 25 m radius. Samples of the O horizon associated with the profiles were obtained using 25 × 25 cm plots, and the sublayers Oi, Oe, Oa were identified, considering their morphofunctional properties [64, 65]. Biomass was determined on samples dried at 44 ± 1 °C for 72 h at constant weight. Samples were ground (Gold enwall model electric mill), sieved to 0.25 mm, dried at 105 °C for 72 h, and ashed at 550 ± 1 °C for 4 h [66, 67] in a porcelain crucible (Thermolyne Furnace III type 19300 Controller). Loss on ignition (LOI) was determined by weight difference with an analytical balance (± 1 mg; RADWAG AS 160/X). The SOC content was calculated using the following equations:

$$LOI,\%=\frac{Sample weight \left(g\right)105^\circ C-Weight \left(g\right)550^\circ C}{\text{Weight }105^\circ \text{C}} \times 100$$

The OC content of the organic layer was determined in accordance by Campos [67].

$$SOC{\text{LECO}}\text{, \%= }-\text{2.901 + 0.547}\left(LOI\right)$$

where: *LOI* = *Loss-on-Ignition*.

The carbon stock and the physical and chemical characterization of the soil were assessed from a soil profile for each of the seven sites, according to FAO [63], Schoeneberger et al. [68], and Siebe et al. [69]. Each soil horizon was described, and 1.5 kg of sample was taken from each horizon. The samples were dried at room temperature, ground, and sieved at 2 mm and 0.25 mm [70, 71].

### Calculation of SOC content and stock

Soil organic carbon (SOC) content was determined by the wet oxidation method with potassium dichromate and its titration with ferrous sulfate [70]. The bulk density was determined by the cylinder method. The depth of the horizons considered the thickness of the genetic horizons of the soil, and the rocks stoniness (%) was determined in volume on the face of the horizons. The carbon stock was determined using the following equation. [56, 71, 72]:

$$SOC (Mg/ha)=\rho s.\frac{SOC}{1000} . d(1-\frac{G}{100})$$

$\rho$_s_ = Bulk density kg m^−3^, d = Horizon depth in m, SOC (%) G = rock fragments (%).

### Soil properties

Soil properties were characterized for each horizon. Properties determined included the pH ratio 1:2.5 water/soil, texture, carbon and nitrogen content, C/N according to Van Reeuwijk [70], and bulk density (BD) [71].

### Statistical analysis

To evaluate the relationship of the study sites in terms of their community attributes and properties of the surface and subsurface soil horizons, with the highest organic carbon content, a principal component analysis (PCA) was performed [73]. A data matrix of 10 variables was constructed for the seven study sites. The variables considered were related to plant diversity and structure (richness, diversity, stem density, basal area, aboveground biomass) and edaphic properties (bulk density, pH, organic carbon, total nitrogen, C:N ratio) of the sites. In the case of edaphic variables, the average values of bulk density, pH, total nitrogen, and C:N ratio of the first 30 cm of soil depth were considered, and the accumulated OC in the litter as the sum of OC content of each organic layer (Oi, Oe, Oa). The variables used in the different units and scales were standardized prior to the analysis.

From the groups resulting from PCA, the carbon stocks of the different pools were compared through a generalized linear model, considering the specific site and the different pools like factors, with a normal distribution and an identity linkage function. Between-pool comparisons were performed using the Bonferroni test. All analyses were performed with the Jamovi program, version 1.1.9 (The Jamovi Project, 2020).

## Results

### Composition and structure of plant communities

A total of 51 woody species belonging to 18 families were recorded. Lauraceae and Fagaceae were the families with the highest number of species, with six and five, respectively. Community richness ranged from 14 to 22 species between communities and diversity values were low to medium (1.8–2.3); the sites located at 1254 and 1115 m asl recorded the highest richness and diversity (Table 1). The similarity between the communities is low, as they share less than 30% of species; those located at 1254 and 1295 show the highest similarity (41%, Table 2).

**Table 1:** Diversity and structure of the woody communities of mountain cloud forest

Elevation (m asl)	Richness	H´	Density (Ind ha^−1^)	Basal aérea (m^2^ ha^−1^)	Aboveground biomass (Mg ha^−1^)
1115	19	2.3	1690	35.36	186.86
1199	16	1.6	3590	22.93	89.72
1235	18	1.8	3610	24.01	85.75
1238	22	2.3	1630	21.67	83.03
1254	17	1.9	1660	30.7	139.33
1263	16	2.0	1800	47.44	238.36
1295	14	1.9	840	31.9	154.32

**Table 2:** Similarity (Jacard’s index) between woody communities of mountain cloud forest

Elevation (m asl)	1115	1199	1235	1238	1254	1263
1115						
1199	0.32					
1235	0.20	0.24				
1238	0.29	0.28	0.22			
1254	0.28	0.22	0.25	0.40		
1263	0.18	0.25	0.16	0.24	0.26	
1295	0.25	0.20	0.18	0.23	0.41	0.29

Structurally, the communities were generally heterogeneous, with two of them having the highest basal area and biomass values (1263 and 1115 m asl; Table 1); while the communities located at 1238 and 1291 m asl had average values and the remaining three communities had the lowest values (1199, 1235, 1254 m asl; Table 1). Stem density ranged from 840 to 3610 individuals ha^−1^, with the highest numbers in the communities at 1199 and 1235 m asl (Table 1).

**Fig. 2:**
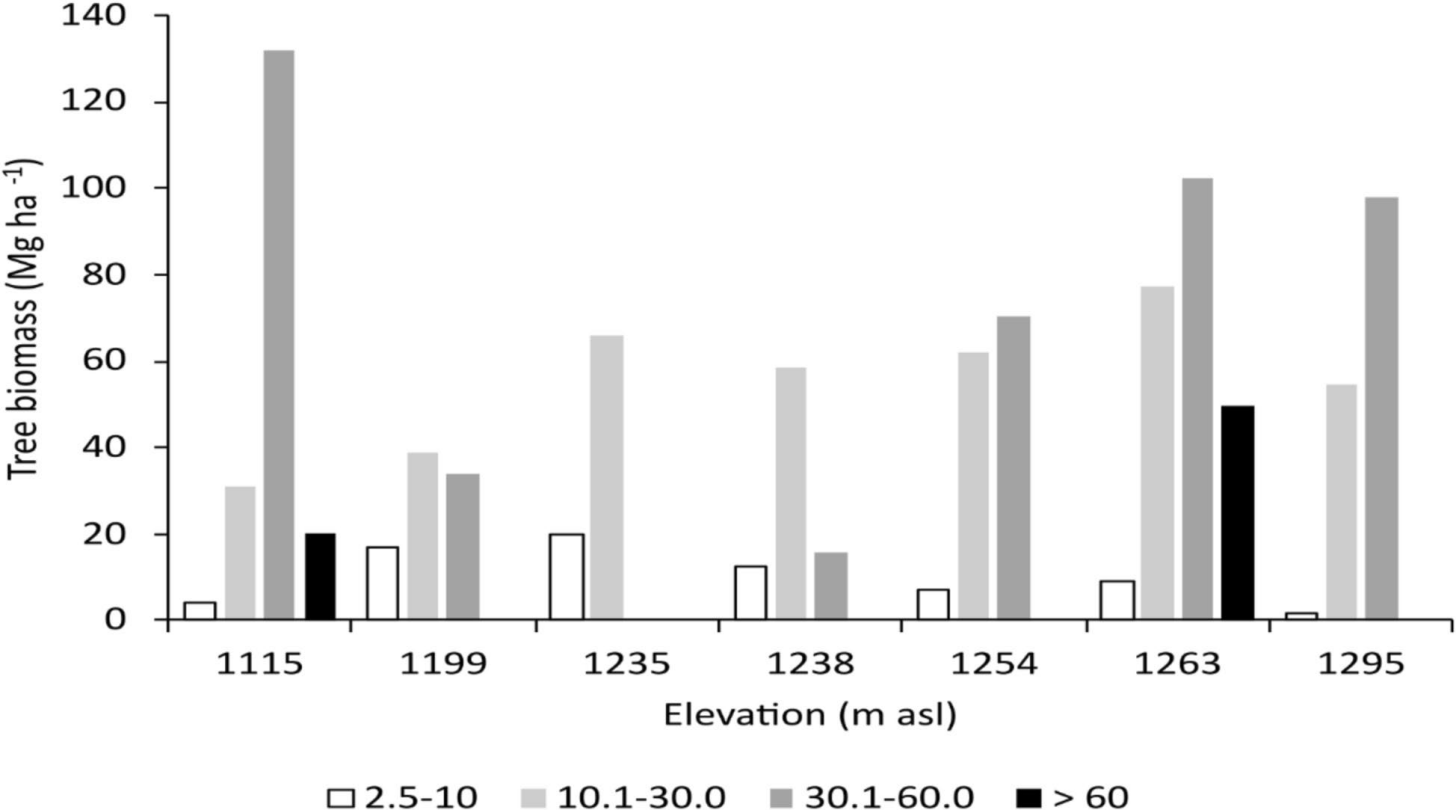
Contribution of diameter classes to the above-ground biomass of cloud forest communities in the micro-basin of Valle Verde—Ojo de Agua de San Francisco, Jalpan de Serra, Querétaro

The structural variation of the communities was associated with above-ground biomass. The sites with the lowest above-ground biomass (1199, 1235, 1238 m asl) had the lowest basal area values and the highest density values, contrary to the sites with the highest biomass (1115, 1254, 1263, 1295 m asl; Table 1). The inverse relationship between basal area and stem density suggests that the contribution of diameter classes to above ground biomass varies among communities (Fig. 2). In those with the highest amount of biomass, the diameter classes greater than 30 cm contributed the most (Fig. 2). Among all communities, the diameter class greater than 60 cm DBH is only present at two sites. In contrast, in the communities with lower biomass, the diameter class with the highest contribution was 10–30 cm (Fig. 2). In these sites, the low frequency of individuals in diameter classes > 30 cm is associated with the selective extraction practices performed by the landowners.

### Physical and chemical properties of litter and soil

The study sites presented a sequence of layers differentiated by depth and morphological properties, both in the organic layer and in the mineral soil. The chemical properties of the Oi, Oe/Oe, and Oa layers were slightly acidic (pH 5.9–6.7). The organic carbon content ranged from 360 to 700 g kg^−1^, the contribution of the individual layers was around 50% of the stock at sites 1115, 1119, 1263, and 1291 m asl, while at 1235, 1238, and 1254 m asl, the highest proportion was established in the Oe and Oa layers. The C:N ratio in the litter ranged from 15 to 22 in the Oi and Oe layers, while in the deeper layers (Oa), there was a tendency to increase up to 39 (Table 3).

**Table 3:** Physical and chemical properties of representative profiles mountain cloud forest

Site / depth, cm	Bulk density (g cm^−3^)	Texture			pH (H_2_O) 1:2.5	OC (g kg^−1^)	TN (g kg^−1^)	C:N
		Sand (%)	Silt (%)	Clay (%)				
1115 m asl								
Oi, 3–2.6	–	–	–	–	6.0	270	16.43	16.4
Oe/Oa 2.60–0	–	–	–	–	6.2	550	24.8	22.0
0–6	0.85	45.5	8.25	46.2	7.0	23.5	1.5	15.7
6–17	1.06	23.7	25.0	51.2	7.0	18.6	0.8	23.3
17–25	1.12	33.7	30.0	36.2	7.5	17.0	0.1	15.5
25–36	1.09	40.5	23.2	36.2	6.3	12.0	1.0	17.0
36–61	1.21	48.7	19.9	31.2	6.6	4.0	1.2	10.0
61–72	1.20	63.7	15.0	21.2	6.6	2.9	–	–
72–120	1.20	58.7	15.0	16.2	6.7	2.8	–	–
Site 1199 m asl								
Oi, 8.4 -3	–	–	–	–	6.6	274.1	26.9	22.1
Oe/Oa, 3–0	–	–	–	–	6.8	393.3	25.8	15.2
0–24/26	0.60	33.0	44.3	22.7	5.4	48.0	1.3	36.9
24/26–36/38	1.00	53.7	28.2	18.1	5.6	46.8	1.2	39.0
36/38–57/67	0.95	84.1	4.6	11.3	5.8	6.7	1	33.5
57/67–71/84	1.05	89.8	14.0	3.8	5.9	3.0	0.2	15.0
71/84–94	1.02	87.9	10.3	1.8	5.9	3.0	–	–
Site 1235 m asl								
Oi, 3–5.5	–	–	–	–	5.9	456	242	18.8
Oe, 5.5–3	–	–	–	–	6.3	474	205	23.1
Oa, 3–0	–	–	–	–	6.7	274	0.55	10.7
0–5/15	0.80	10.2	51.3	38.4	5.5	56.1	3.3	17
5/15–20/25	0.60	9.9	43.4	46.7	6.1	46.3	1.2	38
20/25–40/70	0.90	22.3	36.0	41.7	7.0	7.4	0.4	18
Site 1238 asl								
Oi 8–6.4	–	–	–	–	6.6	365	26.5	15
O3, 6.4–3	–	–	–	–	6.8	254	16.9	15
Oa 3–0	–	–	–	–	6.9	274	26.9	39
0–2	0.50	31.9	30.0	38.0	7.2	68.3	3.1	22
2–10/15	0.80	11.9	40	38.0	7.3	58.5	1.6	36
10/15–20/30	0.80	22.0	15.0	73.0	7.3	17.2	0.5	34
20/30–45/50	1.1	10.0	42.0	48.0	7.5	3.0	0.12	25
Site 1254 m asl								
Oi	–	–	–	–	5.9	284	14.2	20
Oe	–	–	–	–	6.3	178	10.5	16
Oa	–	–	–	–	6.2	287	15.5	18
0–5/10	0.47	48	35	16	5.5	14.0	1.3	11
5/10–25/30	1.20	44	32	24	6.4	16.6	0.5	12
25/30–40	0.62	28	34	42	6.9	2.6	0.2	13
40–60	0.79	24	36	40	7.4	2.6	0.2	13
60–80	0.65	32	20	48	7.6	0.68	0.02	34
Site 1263 m asl								
Oi	–	–	–	–	6.5	55	2.24	24
Oe	–	–	–	–	6.7	70.6	18	39
0–2	0.40	50	15	35	6.4	32.0	2.0	16
2–22	0.73	40	20	40	6.2	21.0	1.0	21
22–33	0.97	25	25	50	6.5	15.0	1.0	15
33–43	1.30	20	20	60	6.6	28.0	2.0	14
43–53	1.10	30	15	55	6.7	22.0	–	–
53–60	1.02	20	25	55	6.8	19.0	–	–
60–88	1.01	40.5	30.0	29.4	6.6	2.0	–	–
88–112	–	–	–	–	6.6	1.0	–	–
Site 1295 m asl								
Oi, 5.6–2.5	–	–	–	–	6.9	594	24.0	24
Oe/Oa, 2.5–0	–	–	–	–	6.1	593	29.7	19
0–5	0.64	12	44	44	6.3	41.7	2.04	20
5–16	1.09	8	60	32	7.3	20.0	1.72	12
16–22	1.10	16	60	24	6.9	21.7	1.36	16
22–69	1.13	4	32	64	6.9	8.0	0.40	20
69–97	1.03	8	8	84	6.9	7.7	0.38	20
97–130	0.97	8	8	84	7.4	2.8	0.50	6

*TN* Total nitrogen, *OC* Organic carbon, ratio C:N

Soils from the surface to 40 cm of depth had a low bulk density (< 1 g cm^−3^) and an OC content > 1% (10 g kg^−1^). The underlying horizons had an OC content < 1%, at depths of 70 and 120 cm, in the deepest soils. The C:N ratio varied between 10 and 25 where the total nitrogen content was 0.15–0.53%, particularly in the surface layer up to 30 cm. The pH was slightly acidic to slightly basic in the superficial horizons (up to 40 cm), while between 50 and 120 cm depth, the pH was acidic (pH 5.4–5.9; Table 3).

### Principal component analysis

**Fig. 3:**
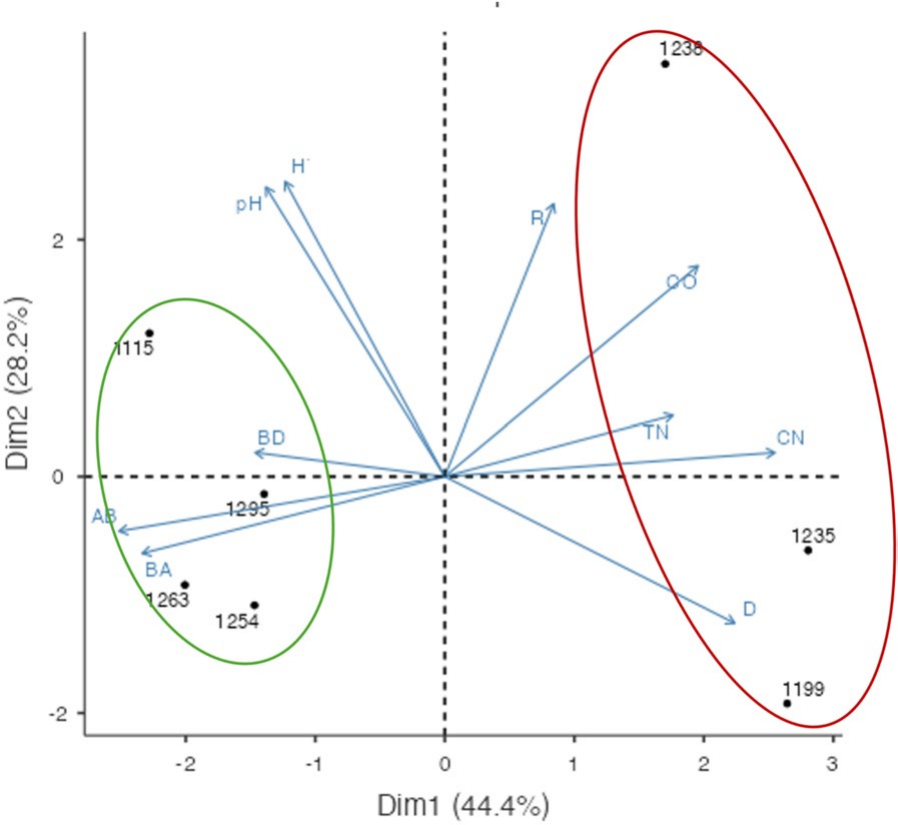
Principal Component Analysis of the communities’ traits and soil variables in cloud forest communities in the micro-basin of Valle Verde—Ojo de Agua de San Francisco, Jalpan de Serra, Querétaro. The direction and size of vectors represents the functional trait weight considered in this study. *R* Richness, *H´* Shanon diversity index, *D* density stems, *BA* basal area, *AB* aboveground biomass, *BD* bulk density, pH, *OC* organic carbon, *TN* total nitrogen, *C:N* ratio carbon–nitrogen

The first two components explained 72.6.2% of the variation of the study sites (component 1 = 44.4% and component 2 = 28.2%; Fig. 3). In the first component, the soil C:N ratio (r = 0.88) and above-ground biomass (r = 0.88) were the most relevant variables in the PCA of communities, while in component two, the most relevant variables were the pH of the A horizon (r = 0.86) and Shannon’s diversity index (r = 0.85). The first group is composed of 4 sites (1115, 1254, 1263, and 1295 m asl), which have the highest values of above-ground biomass and the lowest values of the C:N ratio in the surface soil horizons. The second group consists of three sites (1199, 1235, 1238 m asl), with low above-ground biomass values and high C:N values (Table 3).

### MCF carbon stock

**Fig. 4:**
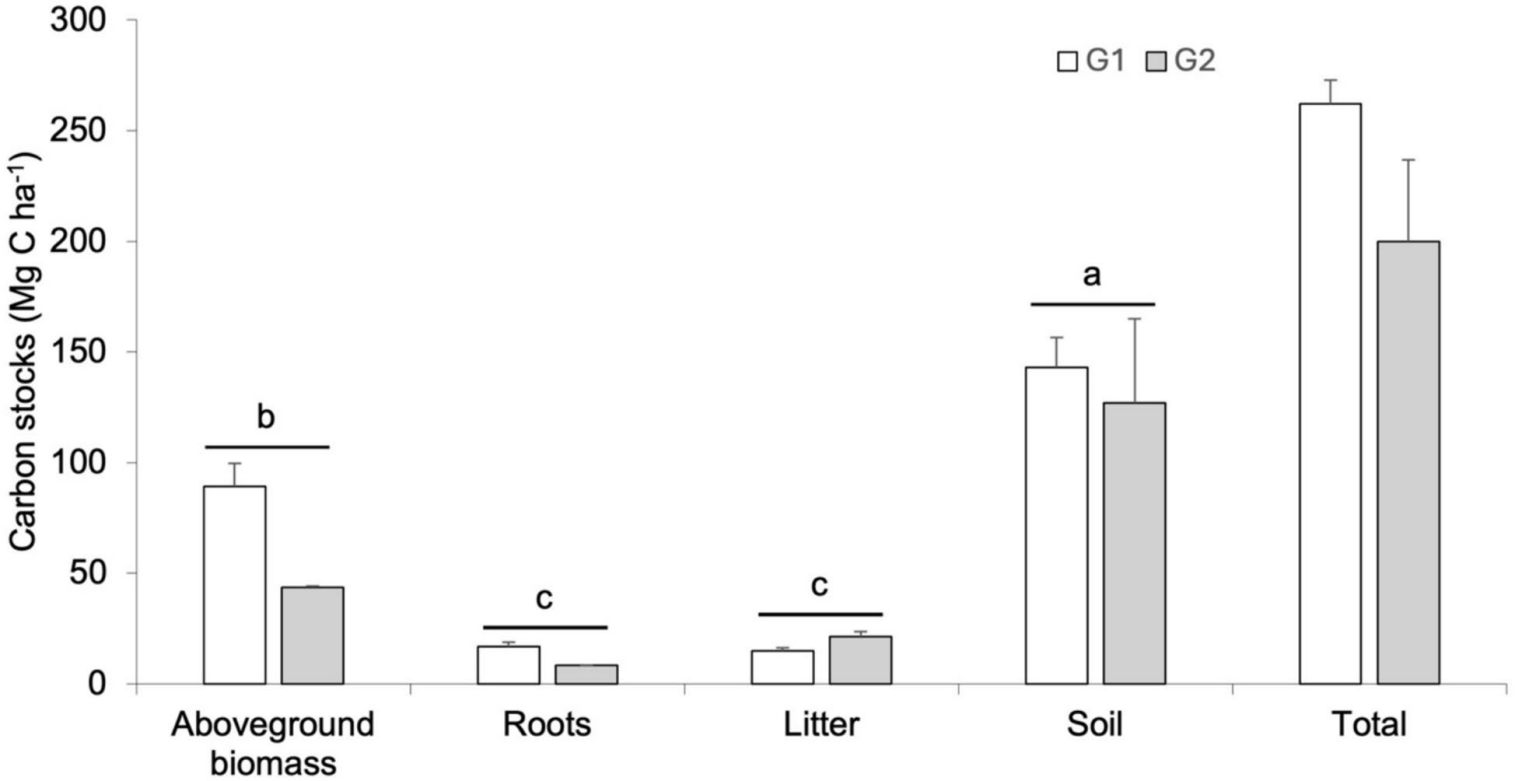
Organic carbon stocks between high (white bars) and low (grey bars) biomass clusters of cloud forest communities in the micro-basin of Valle Verde—Ojo de Agua de San Francisco, Jalpan de Serra, Querétaro. Asterisks above bars indicate significant differences between groups p <  = 0.05

The ecosystem’s carbon stock was established between 145.9 and 279 Mg C ha^−1^, with the highest values recorded in the communities located at 1238 and 1263 m (Table 4). At most sites, soil was the most important pool with more than 50% of the total ecosystem stock; while at 1238 and 1263 m asl its contribution was between 45 and 46%. The second most important pool was above-ground biomass with a contribution between 17 and 42%. Litter and roots were between 5 and 10% of the total ecosystem stock, with litter having the higher contribution (5 and 18%), especially in sites with lower soil stock and above-ground biomass. From the groups generated with the principal component analysis, the contributions of each pool were compared. There were neither significant differences between biomass groups (χ^2^ = 2.89, df = 1, p = 0.09), nor in the interaction between biomass group and reservoir (χ^2^ = 3.99, df = 3, p = 0.262), but there were significant differences between pools (χ^2^ = 107, df = 3, p < 0.001). Soil carbon storage was significantly higher compared to the other pools (Fig. 4); followed by aboveground biomass which was significantly different from the root and leaf litter pools (Fig. 4).

**Table 4:** Distribution of carbon stocks of woody communities of mountain cloud forest

Elevation (m asl)	Above ground biomass	Roots	Litter (Mg C ha^−1^)	Soil	Total
1115	93.43	17.75	13.2	144.3	268.68
1199	44.86	8.52	18.7	198.1	270.18
1235	42.87	8.14	19.3	114.6	184.91
1238	43.51	8.26	26.1	68.08	145.95
1254	77.16	14.66	19.3	119.4	230.52
1263	116.74	22.18	12.9	127.7	279.52
1295	69.66	13.23	14.4	180.0	277.29

### The distribution of the carbon stock in the litter and soil

**Fig. 5:**
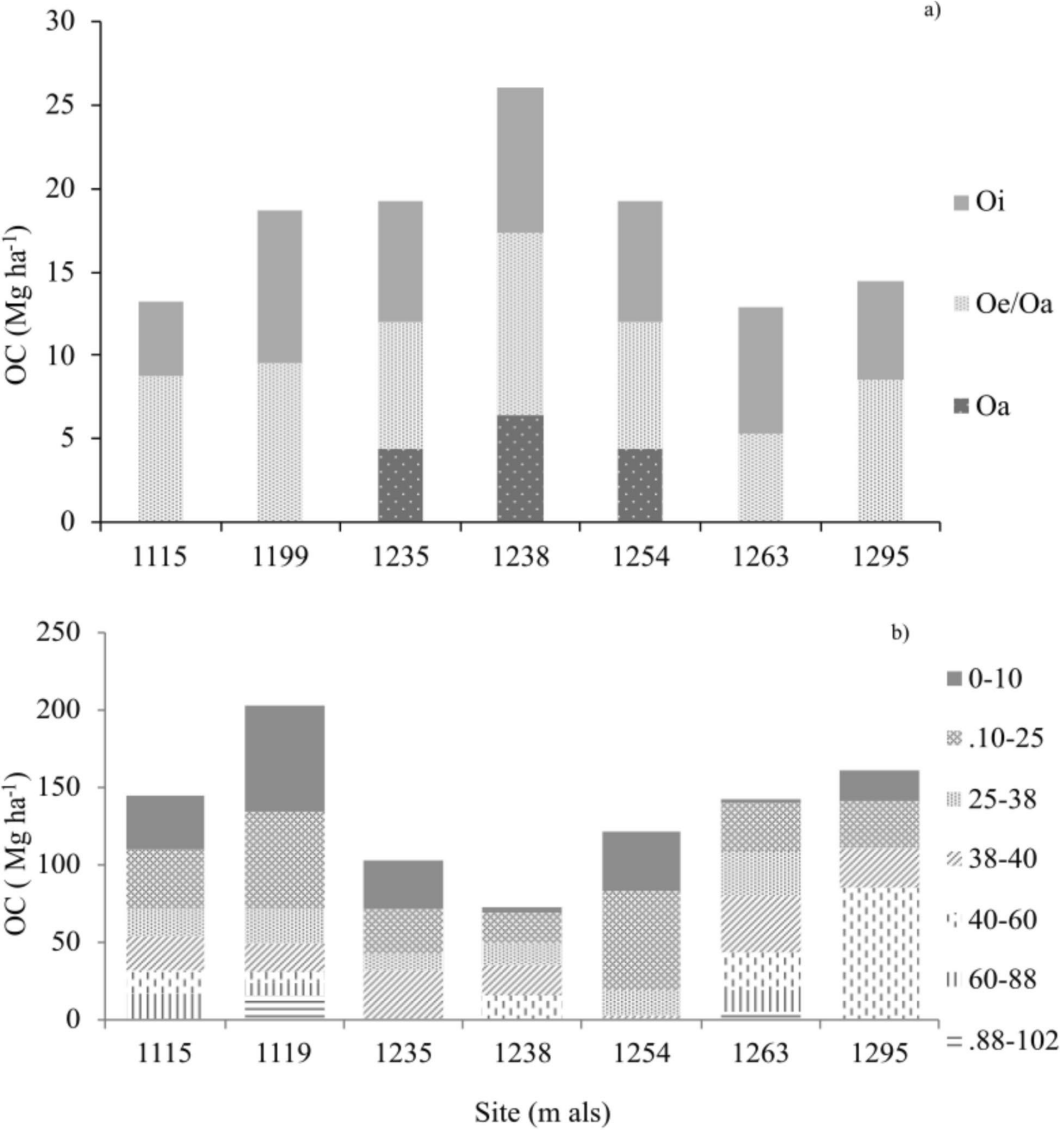
Distribution of carbon stocks in litter (**a**) and soil (**b**) of cloud forest communities in the micro-basin of Valle Verde—Ojo de Agua de San Francisco, Jalpan de Serra, Querétaro

Edaphic C stock was concentrated in two pools, the litter and the soil horizons up to 38–40 cm, where the highest carbon content was established with respect to the total depth (Table 3 and Fig. 5). The O horizon was differentiated into Oa, Oi/Oe, and Oe layers, with a total thickness between 8 and 10 cm. The highest C stock was recorded at the sites at 1238 m asl, with 26.1 Mg SOC ha^−1^, followed by the sites at 1119, 1235, and 1254 m asl with a content of ~ 18 g SOC kg^−1^. The organic layer contribution showed that sites with Oi and Oe/Oa horizons store between 40 and 50% of SOC. Whereas, the sites with better differentiated layers Oi, Oe, and Oa had a higher SOC content, especially Oe and Oa, and their contribution was up to 70%.

The SOC stock was considered at a depth between 40 and 102 cm, where the sites at 1235 and 1254 m asl presented shallower soils, and the sites at 1119 and 1263 m asl were deeper. SOC stock was established between 68 and 198 Mg ha^−1^ at sites 1238 and 1119 m asl, respectively. The higher accumulation of SOC was established in the sequence of horizons up to 38 and 40 cm of depth in the different soils studied. The contribution of this horizon was up to 60% of the total SOC stored in the mineral soil. In the case of sites 1235 and 1254 m asl, the potential SOC stock considered soil horizons at a depth of 30–40 cm, which is the depth of soil development (Table 4 and Fig. 5).

## Discussion

In this study, the carbon stock in different pools in MCF was estimated. A relevant finding was that the contribution of aboveground tree biomass to the overall carbon stock is reduced compared to MCFs in the state of Oaxaca [32]. In the present case, above-ground biomass contributes between 10 and 31% to the carbon stock, while in Oaxaca’s forests, the contribution was greater than 30%. This difference can be related to aspects of anthropogenic management in the plant communities studied, since they are located in an area under agroforestry and livestock management in the RBSGQ [43, 58] and it was possible to distinguish two groups of communities according to their above-ground biomass and its impact on carbon stock.

The values recorded for above-ground biomass carbon stocks potential (42.87–116.74 Mg ha^−1^) were medium to low compared to those recorded in other MCFs, where they ranged from 196.4 to 414 Mg ha^−1^ [2, 64–66]. At the global level, MCFs show a wide variation in their above-ground carbon stock potential**,** ranging from 121. 9 Mg ha^−1^ and 46 to 190 Mg ha^−1^, in Rwanda [74] and Malaysia [75], respectively. In the Americas, stocks of 63.4 Mg ha^−1^ and 384.1 Mg ha^−1^ have been reported in secondary and conserved forests, respectively, in Peru [49]. The low to medium contribution of above-ground biomass to the carbon stock recorded in this study may be associated with forest degradation due to human activities. In tropical rainforests, trees with diameters greater than 60 cm in diameter considerably increase the biomass of the communities; this pattern was described for MCF in Mexico [32]. In our study, trees with diameters greater than 60 cm were only represented in two communities (1115 and 1263 m asl), although they did not contribute the most biomass. In all the communities studied, the 30–60 cm diameter class contributed the largest amount of biomass. The reduction of large trees in the communities studied may be the result of timber extraction that has occurred in the region in recent decades as an economic supplement for the communities [46]. Most of the sites, except for the one located at 1263 m asl (cattle exclusion zone), have visible evidence of timber extraction of trees with large diameter sizes. The PCA provided evidence for a clear separation of the communities according to their above-ground biomass, showing structurally more degraded sites (low basal area and high density of stems in small diameter classes) with less potential for organic carbon stock in this pool. Another structural and diversity characteristic that confirms the level of timber extraction in these forests is the high density of stems, low taxonomic richness and diversity recorded in the communities with the lowest biomass (*e.g.* 1199, 1235 m). In all the sites, the quantities of dead wood are very small (this is why the estimate of necromass was not considered), because it is used as firewood by the villagers [46]. Another factor that may condition the reduction of above-ground biomass is the frequency of forest fires in the last decade [76–78]. Fire incidence leads to changes in the structure and composition of the vegetation. Our results show that the sites with the lowest values of tree biomass (e.g. 1199, 1235 m asl) have the lowest richness and diversity. The presence and abundance of oaks, pines, and liquidambar (e. g. 1263, 1291 and 1253 m asl) have been considered as indicators of fire disturbance due to their fire tolerance, as well as the biomass reduction of up to 50–80% of the aboveground biomass in severe fires [77, 79–81]. In other MCFs, changes in the structure (basal area and composition of plant communities) associated with management intensity influence their carbon stocks potential [48, 80]. The carbon sequestration capacity of forests decreases, with a global reduction of carbon stored in vegetation and soils from 1.99 ± 0.13 to 0.97 ± 0.16 Pg C per year in the last decade. Carbon loss associated with agriculture is estimated at 120.2–373 Tg C per year in the Republic of Congo and Brazil, representing a 34% increase in global loss. Wildfires reduce carbon storage in the surface by approximately 35%, surpassing the effects of logging, which range from 6 to 15% [76]. The conversion of forests to grasslands can lead to a loss of up to 42.5% of the original forest area, causing CO2 emissions of up to 16.3 Mg C ha^−1^ [82].

Organic horizon is another important SOC pool and its production is related to the primary productivity of the vegetation, although its residence time is considered low to medium, depending on the organic structural components, biological activity, acidity, high humidity and low temperature that regulate its transformation [22, 24, 48, 74, 75]. The organic layers in the study sites presented moder/mor and mull/moder type litters, considered as transitional [38, 40] and maintain carbon stock between 12.9 and 18.7 Mg ha^−1^, with an average in the O horizon of 5.04–8.3 Mg ha^−1^. These values are higher than those reported in the MCF of Oaxaca, where the stock capacity was 2.2–4.6 Mg ha^−1^ [32]. This may be related to the sequence of organic horizons with specific biological conditions, where organic structural remnants delimit the morphology [40, 64], so the assessment of the carbon stock is less clear in its morphogenetic definition. World records of litter stock in MCF have shown the importance of this carbon pool. In Rwanda (Africa) values of 3.59 Mg ha^−1^ were recorded [74]; while in Chinese forests they ranged between 0.29 and 4.3 Mg ha^−1^ in undisturbed and disturbed sites; in Malaysia they were of the order of 120 Mg ha^−1^ (mixed soil-litter evaluation) [76]. In the Americas, records show stock between 0.29 Mg ha^−1^ in Peru, 0.36 Mg ha^−1^ in Ecuador, and 4.6 Mg ha^−1^ in México [32, 49].

Sites 1235, 1238, and 1254 m asl had the highest C stock in the organic horizon (7.2 -8.7 Mg ha^−1^, Oi; 7.5- 10.5 Mg ha^−1^, Oe; 4.3- 6.3 Mg ha^−1^, Oa), which was structured as transitional humus type moder/mor, due to the sequence of organic layers Oi, Oe, Oa (Table 3 and Fig. 5). Morphology and carbon stock were related to slow decomposition that allowed layer differentiation, despite its slightly acidic to slightly basic quality (Table 3). The low transformation rate has been related to low CO_2_ production because of the deficiency of aeration and root respiration due to high soil and environmental humidity in MCFs, which decreases decomposition [22, 40]. In addition, it is related to the accumulation of organic residues with high resistance to decomposition inherited from species in the *Quercus* genus and low disturbance processes that favor the differentiation of layers in the O horizon. This process of slow decomposition and differentiation of layer sequences allows the formation of diagnostic folic horizons in MCF soil in Oaxaca [32, 50]. In contrast, sites 1115, 1119, 1263, and 1291 m presented a lower C (3.3 – 9.1 Mg ha^−1^, Oi and 5.3–9.5 Mg ha^−1^, Oe/Oa), and is considered a moder/mull type transitional humus, where the boundary between the layers (Oe/Oa) was diffuse with respect to the clear boundary of the surface layer of the organic horizon (Oi). Reduced C stock and lower litter production are related to the composition of species from which compounds are inherited with lower decomposition resistance and degradation processes [83–85]. Among other reasons are anthropogenic alteration processes due to livestock and the incidence of fire in the area [79]. Surface fires alter the morphofunctional and nutritional properties of the organic layer, which are important for transformation [64, 82]**.** Changes in the organic horizons were observed at sites 1115, 119, 1263, and 1295 m asl, where the low morphological differentiation in the organic horizons Oe/Oa may indicate alterations in the subsurface layers, either due to seasonal changes they actively respond to or management practices like harvest of the forest [83].

The soil organic carbon stock is one of the main pools at the terrestrial level due to stabilization processes resulting from humification and physical protection of organic compounds [86, 87], and the formation of organo-mineral and organo-metal complexes by translocation of organic compounds and clays in sites of high precipitation [25]. In the study sites, soil was the main C pool, particularly for organic C, where soil contributed > 50%, with stocks between 68.0 and 198.1 Mg ha^−1^ (Tabla 4). The C storage potential in the communities studied was found to be within the range reported in other MCFs, which varies from 75 to 200 Mg ha^−1^ in Chiapas, Veracruz, and Oaxaca. [23, 32, 61]. It is also consistent with what has been reported in other MCFs around the world, 159 Mg ha^−1^ in Rwanda [74], 120 Mg ha^−1^ in Malaysia, 118 Mg ha^−1^ (undisturbed soils) in Peru and 252 Mg ha^−1^ in Costa Rica (disturbed and undisturbed soils) [49, 76]. Although sites showed different trends in SOC stocks (Fig. 5), when sites were grouped according to their biomass, organic carbon stocks did not show significant differences, but variations among sites may be associated with the edaphogenetic processes they share that allow carbon stabilization [81, 86].

The vertical distribution of carbon in the study sites shows that accumulation reached the maximum depth of soil development, which varied among the sites from 40 cm to soils 120 cm deep, with the stock ranging from 100 to 200 Mg ha-1 (Table 4 and Fig. 5). The high sequestration potential can also be explained by the translocation of organo-mineral colloids, especially in humid environments with prolonged precipitation [25, 32]. In the soil, the highest carbon stock was found in most sites at a depth of 40 cm, with a range from 13.1 to 49.7 Mg ha-1, accounting for 50% of the carbon accumulated in the soil (Fig. 5). The properties of these layers showed C:N ratios between 10 and 29, slightly acidic to slightly basic pH, and low bulk density (BD) (Table 3), which favors the decomposition of SOM as well as the formation of highly stable organic molecules [32], that restrict decomposition and decrease CO2 emissions [22]. This may be associated with the presence *of Quercus aff. xalapensis, Quercus peduncularis, Quercus germana, Quercus laeta* in the MCF, species that generate organic matter resistant to decomposition [82, 83]. In addition, the presence of high SOC content and storage has been related to physical protection by macro and meso aggregates, as well as by the presence of particulate SOM, which influences soil density [41, 87]. Furthermore, the presence and translocation of humus-clay complexes in soils with high clay content and the dominant presence of humic substances, characteristic of MCF, can be associated with the stabilization of carbon in surface layers [25, 32], promoting the development of deep horizons of 20–38 cm with a high carbon storage potential. (Fig. 5).

The variation in carbon storage among the different communities analyzed can partly be linked to dominant human activities, such as livestock, timber extraction, and fires, which directly affect the soil and serve as indicators of the potential for ecosystem services in MCF [28, 48, 77, 78]. At sites 1254 and 1263 m asl, soils with shallow surface horizons (10 cm deep) and low carbon content (14.0 and 32.0 Mg ha-1, respectively) were considered degraded due to the decrease in carbon content (< 35 g kg^−1^). This results from physical soil degradation processes and increased decomposition of SOM, which subsequently promotes higher CO_2_ production and emissions [21, 22]. It is also influenced by fires, which impact the amount of SOM accumulated in the litter and soil [77, 79, 83–85].

## Conclusions

The carbon sequestration potential of the MCF is found in the soil associated with the organic horizons that develop at the surface and the presence of deep A horizons with high carbon content. The conservation of these layers, despite forest management, reflected in the aerial biomass, demonstrates the resilience of the soil due to carbon stabilization, attributed to the composition of resistant organic compounds and the formation of clay-humus complexes, which reduce the impact of degradation from erosion. This indicates that the conditions of the MCF still maintain the ecological and biogeochemical processes that support carbon sequestration and are regulated because they are within the Sierra Gorda Biosphere Reserve.

The conservation priorities of the MCF management are critically important under both local and federal management plans. Maintaining forest systems with a basal area greater, with minimal extraction and fire impact, helps preserve various carbon pools that play a role in carbon sequestration amid climate change challenges. Additionally, protecting species that are not selectively harvested for firewood could lead to an increase in basal area and biomass, thereby enhancing the importance of aerial carbon storage. Monitoring changes in the carbon reservoirs of the MCF is essential to understand how human activities affect carbon storage, which improves our knowledge of the carbon sequestration potential.

## Data Availability

No datasets were generated or analysed during the current study.

## Abbreviations

MCF: Mountain cloud forests
OC: Organic carbon
SOM: Soil organic matter
MRT: Mean residence time
SOC: Soil organic carbon
RBSGQ: Reserva de la Biósfera de la Sierra Gorda de Querétaro
